# A novel type-2 innate lymphoid cell-based immunotherapy for cancer

**DOI:** 10.3389/fimmu.2024.1317522

**Published:** 2024-03-07

**Authors:** Iryna Saranchova, Clara Wenjing Xia, Stephanie Besoiu, Pablo L. Finkel, Samantha L. S. Ellis, Suresh Kari, Lonna Munro, Cheryl G. Pfeifer, Ladan Fazli, Martin E. Gleave, Wilfred A. Jefferies

**Affiliations:** ^1^ Michael Smith Laboratories, University of British Columbia, Vancouver, BC, Canada; ^2^ Department of Microbiology and Immunology, University of British Columbia, Vancouver, BC, Canada; ^3^ Center for Blood Research, University of British Columbia, Vancouver, BC, Canada; ^4^ The Djavad Mowafaghian Center for Brain Health, University of British Columbia, Vancouver, BC, Canada; ^5^ Department of Medical Genetics, University of British Columbia, Vancouver, BC, Canada; ^6^ Department of Zoology, University of British Columbia, Vancouver, BC, Canada; ^7^ Department of Urologic Sciences, University of British Columbia, Vancouver, BC, Canada; ^8^ The Vancouver Prostate Centre, University of British Columbia, Vancouver, BC, Canada

**Keywords:** cell-based cancer immunotherapy, type 2 innate lymphoid cells, ILC2, interleukin-33, adoptive cell transfer, tumor infiltrating lymphocytes, ILC2 heterogeneity, type-1 immunity of ILC2

## Abstract

Cell-based cancer immunotherapy has achieved significant advancements, providing a source of hope for cancer patients. Notwithstanding the considerable progress in cell-based immunotherapy, the persistently low response rates and the exorbitant costs associated with their implementation still present a formidable challenge in clinical settings. In the landscape of cell-based cancer immunotherapies, an uncharted territory involves Type 2 innate lymphoid cells (ILC2s) and interleukin-33 (IL-33) which promotes ILC2 functionality, recognized for their inherent ability to enhance immune responses. Recent discoveries regarding their role in actuating cytolytic T lymphocyte responses, including curbing tumor growth rates and hindering metastasis, have added a new dimension to our understanding of the IL-33/ILC2 axis. These recent insights may hold significant promise for ILC2 cell-based immunotherapy. Nevertheless, the prospect of adoptively transferring ILC2s to confer immune protection against tumors has yet to be investigated. The present study addresses this hypothesis, revealing that ILC2s isolated from the lungs of tumor-bearing mice, and tumor infiltrating ILC2s when adoptively transferred after tumor establishment at a ratio of one ILC2 per sixty tumor cells, leads to an influx of tumor infiltrating CD4+ and CD8+ T lymphocytes as well as tumor infiltrating eosinophils resulting in a remarkable reduction in tumor growth. Moreover, we find that post-adoptive transfer of ILC2s, the number of tumor infiltrating ILC2s is inversely proportional to tumor size. Finally, we find corollaries of the IL-33/ILC2 axis enhancing the infiltration of eosinophils in human prostate carcinomas patients' expressing high levels of IL-33 versus those expressing low levels of IL-33. Our results underscore the heightened efficacy of adoptively transferred ILC2s compared to alternative approaches, revealing an approximately one hundred fifty-fold superiority on a cell-per-cell basis over CAR T-cells in the specific targeting and elimination of tumors within the same experimental model. Overall, this study demonstrates the functional significance of ILC2s in cancer immunosurveillance and provides the proof of concept of the potential utility of ILC2 cell-based cancer immunotherapies.

## Introduction

The advent of cancer immunotherapies based on the demonstration that T Lymphocytes limit the growth of tumors has revolutionized the available treatment landscape for several malignancies (1). Initially, the focus was on exploiting autologous tumor-infiltrating lymphocytes (TILs) for their inherent anti-tumor activity, which evolved into the development of genetically engineered Chimeric Antigen Receptor (CAR) T-cell therapies targeting specific tumor antigens (2–5). However, the efficacy of CAR-T-cell therapies in solid tumors remains limited, often hampered by challenges such as insufficient tumor infiltration, immune checkpoint ligand expression, and immunosuppressive tumor microenvironments. Additionally, issues including antigen specificity, tumor heterogeneity, logistical complexities, and cytokine release syndrome further restrict their application (6–16). This shift has prompted the exploration of alternative cell-based therapeutic approaches, with innate lymphoid cells (ILCs), especially type 2 ILCs (ILC2s), emerging as potential candidates (17).

ILC2s, an integral part of the ILC family, are critical for orchestrating various immune responses. They lack antigen-specific receptors but are known for their versatility and ability to produce a range of cytokines in response to environmental cues. Predominantly, ILC2s are identified by their expression of the transcription factor *GATA3* and their production of Th2 cytokines like IL-4, IL-5, IL-9, and IL-13 in response to signals such as IL-33, IL-25, and TSLP. This cytokine production is central to their role in various physiological processes, including tissue repair, metabolic homeostasis, and the defense against helminthic infections (18, 19). Moreover, ILC2s are implicated in the pathogenesis of several allergic diseases, including asthma, rhinitis, and atopic dermatitis, where their dysregulated activity contributes to disease progression (20). The distribution of ILC2s across different tissues, including the lung, skin, gut, and adipose tissue, is notable for its impact on their functional characteristics. In each of these environments, ILC2s display unique phenotypic traits and respond distinctively to local stimuli. For instance, in the lung, ILC2s are involved in airway hyperreactivity and mucus production, while in the gut, they play a role in maintaining epithelial integrity and responding to dietary antigens (20). Additionally, ILC2s display migratory ability under healthy and inflammatory conditions, allowing them to mobilize to sites in response to activation signaling such as IL-33 release (21). This tissue-specific and migratory functionality of ILC2s highlights their adaptability and importance in maintaining local and distal homeostasis.

In the context of cancer, the role of ILC2s is multifaceted and complex. On one hand, there is evidence suggesting their involvement in tumor-promoting processes. For instance, in certain solid tumors, such as bladder cancer, ILC2s can contribute to the creation of an immunosuppressive environment, facilitating tumor immune evasion and growth by interacting with myeloid-derived suppressor cells (MDSCs) (22–25). This pro-tumorigenic aspect of ILC2s is mediated through their secretion of type 2 cytokines, which can promote tumor progression and metastasis (22–25).

Conversely, emerging evidence indicates a potential anti-tumor role for ILC2s, particularly influenced by the tumor microenvironment. In the presence of IL-33, ILC2s can enhance anti-tumor immunity by upregulating MHC-I expression on tumor cells and improving their recognition and elimination by cytotoxic T lymphocytes. We initially observed this phenomenon in tumor models where the role of ILC2s appears to be significantly modulated by the presence or absence of IL-33 (26–28). In these models, the interaction between ILC2s and the tumor microenvironment, especially the cytokine milieu, plays a crucial role in determining their pro- or anti-tumoral effects. Overall, our earlier research established, for the first time, that ILC2s play a crucial role in supporting Th1 responses by promoting interactions between the innate and adaptive components of the immune response.

This dual nature of ILC2s in cancer raises intriguing possibilities for their therapeutic application. However, the therapeutic application of ILC2s is not without challenges (28). The phenotypic plasticity of ILC2s, influenced by their tissue environment, raises questions about their stability and efficacy in a therapeutic setting (29). Additionally, the potential for off-target effects, including exacerbation of allergic responses or promotion of tumor growth in certain contexts, must be carefully considered. Furthermore, the development of strategies to selectively activate or suppress ILC2 functions in the tumor microenvironment is crucial for their successful application in cancer therapy (30). This necessitates a deeper understanding of the signaling pathways and environmental factors that regulate ILC2 activity.

In addition to these biological considerations, there are practical challenges in developing ILC2-based therapies. These include optimizing the methods for isolating, expanding, and engineering ILC2s for therapeutic use (31). Given that ILC2s do not possess antigen-specific receptors like T lymphocytes, strategies to enhance their tumor-targeting capabilities and to ensure their persistence and functionality in the tumor microenvironment must be developed. This might involve genetic engineering approaches or the use of bi-specific antibodies or cytokines to direct ILC2s to tumor sites.

Comparing potential ILC2 cell-based therapies with other cell-based strategies such as NK cell therapy offers valuable insights. NK cell therapies have shown promise, particularly in targeting hematologic malignancies and some solid tumors. However, they face challenges similar to those of CAR T-cell therapies, including issues with tumor infiltration, persistence, and specificity (32–34). ILC2s, with their unique cytokine profiles and ability to modulate the tumor microenvironment, could potentially overcome some of these limitations. Their role in both promoting and suppressing tumor growth, depending on the context, suggests that they could be engineered or modulated to exert a more controlled and targeted anti-tumor effect. Given these complexities, the potential of ILC2-based therapies in cancer treatment remains unresolved. However, ongoing studies are exploring the mechanisms underlying the dual role of ILC2s in cancer, their interactions with other immune cells in the tumor microenvironment, and the ways in which they can be harnessed for therapeutic purposes. These studies will be crucial in determining the feasibility and efficacy of ILC2-based therapies in clinical settings.

Our previous work demonstrated that mice genetically lacking ILC2s have a reduced capacity to impede tumor growth *in vivo;* providing the first evidence that ILC2s participate in cancer immune surveillance (26). Therefore, the exploration of ILC2s in the context of cancer therapy may provide new avenues for the development of innovative immunotherapies. By harnessing the unique properties of these cells, it may be possible to overcome the limitations of current therapies and thereby develop more effective treatments for both solid and hematologic tumors. However, a comprehensive understanding of the biology of ILC2s, including their role in sculpting the tumor microenvironment is required to overcome the challenges associated with calibrating their therapeutic applications. Here we investigate the therapeutic potential of adoptively transferred ILC2 cells in cancer, with the potential outcome of offering new strategies to treat cancers resistant to current therapies.

## Results

### Cytotoxic T lymphocytes and T helper cells are critical for anti-tumor immune response *in vivo*

**Figure 1:**
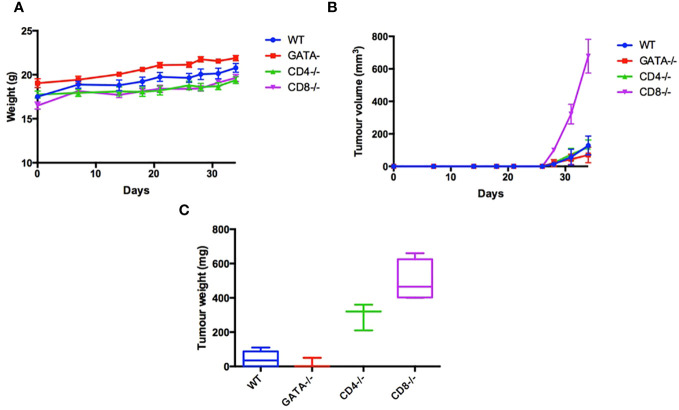
Characterization leukocyte populations that contribute to immune response to the TC-1 tumor *in vivo*. To examine the immunological characteristics of the TC-1 cell line *in vivo*, 5x10^5^ cells were subcutaneously injected into the right flank of 32 mice: C57BL/6 (n=8), GATA-/- (n=8), CD4-/- (n=8), and CD8-/- (n=8). **(A)** Body weight was recorded three times a week until humane end point. **(B)** Tumor volume was measured three times a week (V= Length x Width x height X pi/6). **(C)** After 34 days all mice were euthanized, and tumor weights were measured. Outliers were removed if two SEM outside the average calculated for each group.

As an initial step, we sought to determine if cytotoxic T lymphocytes (CTL) and T helper cells, as well as eosinophils, are required to inhibit the growth of TC-1 cells, a murine lung tumor model derived from C57BL/6 mice. To determine which additional leukocyte populations besides ILC2s contribute to the immune response to the TC-1 tumor *in vivo*, 5x10^5^ TC-1 cells were subcutaneously injected into the right flank of a variety of 6–8-week-old, synergistic mice. To assess if mice lacking CTLs (CD8^-/-^, n=8), or T helper cells (CD4^-/-^, n=8) or eosinophils (*GATA1^-/-^*, n=8) were able to affect the growth of tumors, animals with individual genetic ablations of these three subpopulations of leukocytes were inoculated with TC-1 tumour cells. A control group, with a fully capable immune system (wild type C57BL/6 mice, n=8), was also included. Animals inoculated with the TC-1 cells were monitored for tumor growth. All mice gained weight at a healthy rate with no significance difference between any of the four groups (**Figure 1A**). Of the four groups, mice lacking CTLs developed the largest tumors in comparison to the wild type controls (**Figures 1B, C**). This demonstrates that the CTLs play a crucial role in recognizing the TC-1 cells and reducing overall tumor burden. The mice genetically lacking T helper cells also showed a more significant tumor volume than the wild type control (**Figures 1B, C**). Interestingly, mice lacking eosinophils (*GATA1^-/-^*) also have a trend towards a reduced tumor burden in comparison to the control group, however, this difference was not found to be statistically significant. In conclusion, modalities that increase CD8+ and CD4+ T lymphocyte responses, including the protein products produced by innate immune cells, such as eosinophils and ILC2s, may increase anti-tumor responses and reduce tumor growth.

### The frequency of ILC2s is elevated in primary tumors expressing IL-33, when compared to metastatic tumors with lack of IL-33 expression

**Figure 2:**
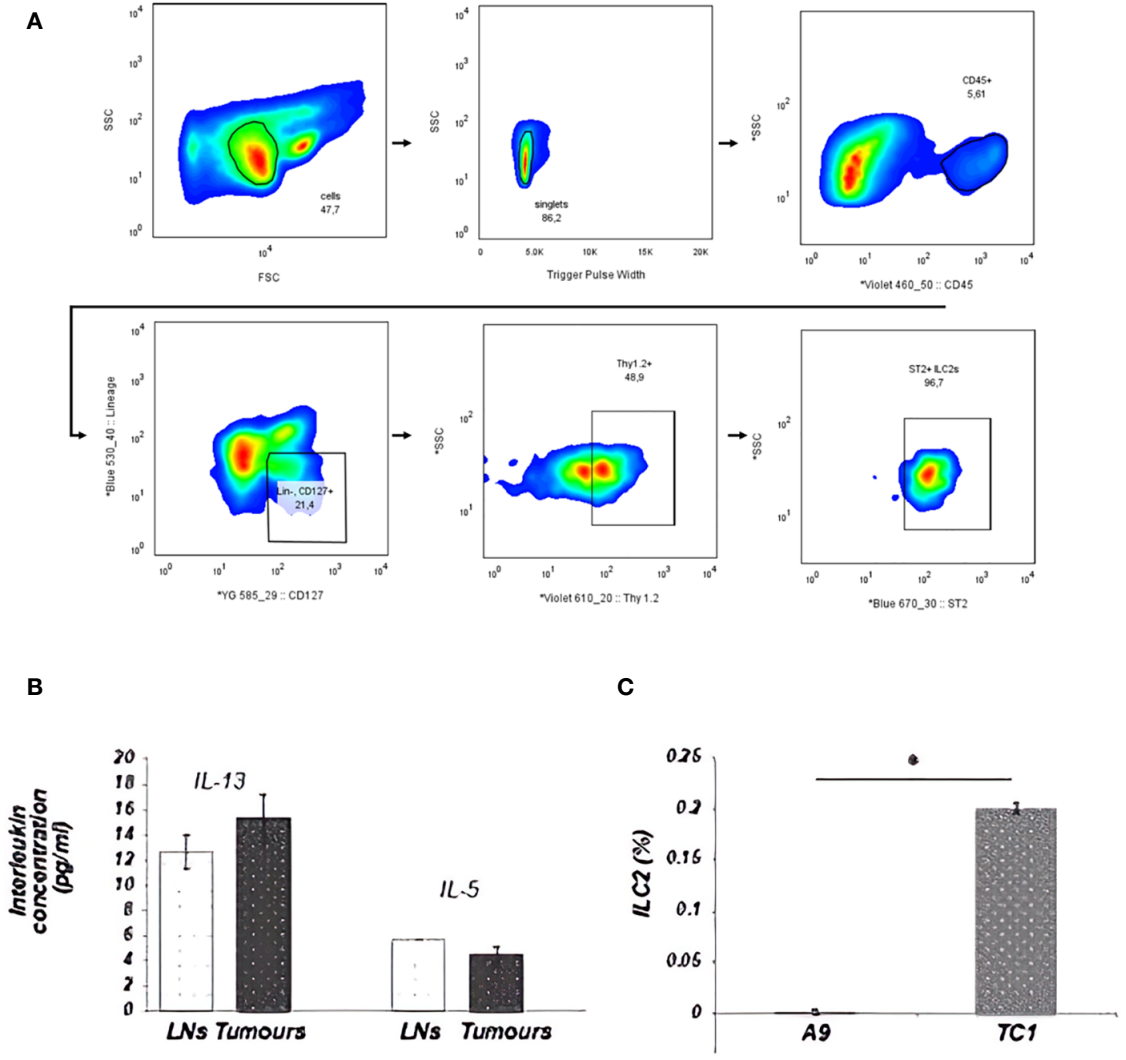
ILC2s from tumors were sorted by FACS as Lin-ST2+CD127+CD90.2+ cells. A total of 2x10^5^ cellular events from disaggregated tumors were used to create a profile for each tumor. **(A)** Illustration of the ILC2 gating strategy used for all ILC2 isolation in this study. Cells were first enriched with EasySep Mouse ILC2 Enrichment Kit (STEMCELL Technologies) then sorted from lungs by FACS as Lin−, CD45+, ST2 +, CD127, + Thy1.2+ cells. Sequential gating strategy is based on cell size [P1: small and non-granular cells, forward scatter (FSC) versus side scatter (SSC)], depletion of doublets (P2: SSC vs. Trigger Pulse Width), and selection of CD45 + cells. Cells with lineage- related markers were rigorously depleted during enrichment and isolation. **(B)** ILC2s, isolated from tumors, appeared to be fully functional and retained the ability to secrete IL-13 and IL-5 cytokines. This graph shows ILC2s, which were isolated from either disaggregated draining LNs, including mesenteric, inguinal and lumbar LNs, or from TC-1 tumors. **(C)** The percentage of ILC2 cells isolated from total cells in the tumor is represented in the bar graph. The number of ILC2s that could be isolated from the tumor went up in direct relation to the ability of the tumor cells to secrete IL-33. This difference was statistically significant between the number of ILC2 cells isolated from the primary TC-1 and metastatic A9 tumors (*P<0.05; Student’s t-test). The ranges represent the data from animals within each tumor group, where LNs (n = 8 animals) and tumors (n = 4 animals). * indicated significance.

The development and function of ILC2s from the lungs are classically dependent on IL-33, though ILC2s isolated from different organs and tissues can be activated by other mediators. The difference in IL-33 expression between primary and metastatic tumors enabled examination of the involvement of ILC2s and IL-33 in cancer progression. First, the presence of ILC2s in the disaggregated tumor tissue was detected using flow cytometry. ILC2s were identified as cells that did not express leukocyte-lineage cell surface markers (Lin: CD3, CD4, CD8, TCRβ, CD19, CD11c, Gr-1, NK1.1, Ter119), while exhibiting a distinct pattern of cell-surface marker expression of the IL-33 receptor (ST2) chain, IL-7 receptor subunit IL-7Ra (CD127) and Thy1.2 (CD90.2) (**Figure 2A**). The population of Lin-ST2+CD127+CD90.2+ cells was further shown to be morphologically similar to lymphocytes: round in shape with a high nuclear to cytoplasm ratio. Upon isolation, this cell subset was able to secrete IL-5 and IL-13 cytokines after 3h stimulation *ex vivo*, as described by Halim et al. (35) (**Figure 2B**). Further validation of ILC2s identity that remain Lin- after culturing is shown in **Supplementary Figures 1**, **2**. These data suggest that the population of Lin- ST2+CD127+CD90.2+ cells detected in tumors were phenotypically and functionally ILC2s. Next, the level of ILC2 infiltration into primary IL-33 expressing (TC-1) or metastatic IL-33 low (A9) tumors was assessed. A significant decrease in the numbers of ILC2s within the disaggregated tissues of metastatic (A9) versus syngeneic, antecedent primary (TC-1) tumors was observed (**Figure 2C**). These data suggest that ILC2s can be activated by IL-33 released by the tumors and are involved in immune surveillance towards tumors.

### Products secreted by ILC2s isolated from lungs of tumor-bearing (tILC2s) animals have decreased type 2 identity, when compared to naïve ILC2s (nILC2s)

**Figure 3:**
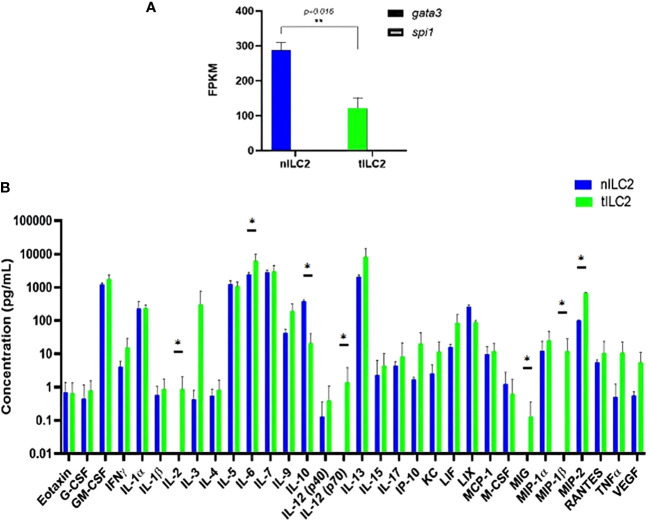
tILC2 cells lose type 2 identity and produce more pro-inflammatory mediators than conventional nILC2s *(ex vivo).* **(A)** At the RNA level, purified ILC2s from WT and tumor-bearing mice express the gene for GATA-3, but not PU.1, which is the master regulator for myeloid fate. The GATA-3 gene is also significantly down-regulated in tILC2s compared to nILC2s. FPKM = fragments per kilobase million. **(B)** A 32-Plex mouse cytokine and chemokine array from Eve Technologies for day 3 culture supernatants of nILC2s and tILC2s. tILC2s secrete significantly higher amount of pro inflammatory cytokines (p<0.05) compared to nILC2s. Error bars represent standard error of the mean and experiment n=3. Statistical tests per cytokine are unpaired *t*-tests. “*” and “**” indicates significance.

To understand how adoptively transferred ILC2s derived from TC-1 tumor-bearing donor mice (tILC2s) reduce tumor growth and to assess the effects of tumors on the transcriptional and translational profile of ILC2s, we conducted multiple studies on RNA and protein expression levels. The bulk RNA sequencing analysis confirmed *GATA3* positive gene expression in both ILC2 groups: naïve (nILC2s) and tILC2s (36, 37). However, the *GATA3* gene was significantly down-regulated in tILC2s compared to nILC2s. The expression of *Spi1* gene, which encodes the master regulator *PU.1* that determines myeloid development (38), was not detected (**Figure 3A**). Our study (39) also highlighted the decreased type 2 identity of tILC2s through scRNA sequencing during tumor progression, as *GATA3* gene expression decreases due to the presence of growing tumor in a mouse body. Overall, it appears that the neoplastic environment suppresses the expression of *GATA3* signaling pathway in ILC2 cells affecting phenotype/functionality of tILC2s accordingly.

Second, to assess if transcriptional changes were reflected at the protein level, we examined protein arrays of culture supernatants. The results from these protein arrays highlight the functional plasticity of ILC2s. One of the most significant shifts was detected in IL-12 expression during the analysis of the log_10_ concentration of 32 cytokines secreted by tILC2s and nILC2s in culture supernatants (**Figure 3B**). We found that the full length of biologically active IL-12 (p70) was secreted by tILC2s, but not by nILC2s. Given that IL-12 signaling pathway has been confirmed, as a driving mechanism for cytokine production plasticity in human ILC2 (40, 41), we suggest that IL-12 might provide the paracrine interactions associated with immune response during tumor development. It has been previously shown that IL-12 signaling promotes the effector function of CD8^+^ T cells, when IL-33 synergizes with the TCR (42).

We also detected that tILC2s produce higher level of pro-inflammatory modulators, such as TNFα, IL-6, MIG (CXCL9), MIP-1β (CCL4) and MIP-2 (CXCL2), which act in the early phases of the innate response (43). For example, MIP-2 affects immune cells recruitment and activation through the p38 MAPK-dependent signaling pathway (44). CXCL9 is an IFN-γ induced chemokine, and it has the potential to provide amplification of the IFN-γ signal (45). Additionally, the level of IL-2 is significantly increased in tILC2. Not only does IL-2 act as a signal for cell proliferation, but it is also an important activator of CTLs. At the same time, tILC2s express lower level of the immune-inhibitory cytokine IL-10. These changes have the potential to expand adaptive immune responses via context-specific paracrine interactions.

Overall, these results demonstrate that the presence of a growing tumor in the organism induces the plasticity of ILC2s. Thus, while nILC2s maintain their conventional identity, the gene and protein expression profiles of tILC2s seems to be shifted to an inflammatory phenotype. Notably, this plasticity has the possibilities to expand adaptive immune responses via context-specific stimuli and requires future experimentations.

### Intraperitoneal injection of IL-33 increases the amount of tILC2s in both the lungs and tumors, and decreases the tumor size via IL-33/ILC2 axis

**Figure 4:**
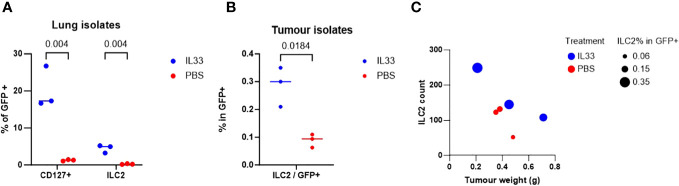
TC-1-bearing GFP+ mice treated with IL-33 yielded higher amount of ILC2s. **(A)** Percentage of lin-GFP+CD45+CD127+ total ILCs isolated from lung tissues are significantly increased with IL-33 *in-vivo* treatment (multiple unpaired t-tests). **(B)** Percentage of ILCs in total tumor-infiltrating GFP+ cells is significantly increased with IL-33 treatment (paired t-test). TC-1 tumors are GFP negative, therefore all GFP+ cells are host cells. **(C)** Mice treated with IL-33 showed inverse relationship between ILC2 count vs tumor size. More ILC2s are detected in the smaller sized tumors. This relationship is not observed in PBS control group.

We examined the *in vivo* effect of IL-33 activation on tILC2s in tumor-bearing mice in terms of their overall yield, the ability to infiltrate tumor mass, and their effect on the tumor weight. In this study, GFP-negative TC-1 tumors were established in C57BL/6-Tg(CAG-EGFP) mice with widespread expression of EGFP. The mice were then injected intraperitoneally (i.p.) with IL-33 or PBS (control). EGFP+ tILC2s were sorted from lungs and tumors (**Supplementary Figures 3**, **4**). After IL-33 i.p. injection, host mice revealed a significant increase (multiple unpaired T-test) in the percentage of tILC2s in gated GFP+ cells isolated from the lungs (**Figure 4A**), indicating a robust activating effect of IL-33 in promoting the expansion of tILC2s in the lung tissues. Furthermore, we also found significant increase of tILC2s within the TC-1 tumor mass (**Figure 4B**). The established tumors do not express GFP, therefore, any GFP+ cells detected within the tumors should be of host origins. With GFP positivity and other ILC2-related markers, we can detect the tILC2s presence within the tumors. One of the most intriguing findings was the discovery of an inverse correlation between the number of tILC2s and the size of the tumors in the IL-33-treated group (**Figure 4C**). Remarkably, the smaller-sized tumors exhibited a higher number and frequency of tILC2s compared to the larger-sized tumors. This phenomenon was exclusive to the group of IL-33-injected mice and was not observed in the control group. This inverse correlation between the numbers of tILC2s and tumor size suggests a potential regulatory role of tILC2s in immune surveillance during tumor development, possibly indicating that an increased presence of tILC2s may have a suppressive effect on tumor growth. This finding highlights the intricate interplay between the immune response, cytokine content, and tumor progression, providing valuable insights for further exploration in the field of cancer immunotherapy.

### Adoptive transfer of tILC2s mediates cancer-free survival

To explore the potential of tILC2 cells in mediating protective anti-tumor immunity and establishing a proof-of concept for ILC2 cell-based immunotherapies in general, we assessed the impact of the adoptive transfer of tILC2 cells into animals with harboring tumors. This involved transferring activated tILC2 cells isolated from donors into recipient animals with established primary tumors to examine the effect on solid tumor growth *in vivo*. To prime tILC2-donor mice, TC-1 tumor cells were first injected subcutaneously (s.c.) into the right flank of C57BL/6 mice. Approximately three weeks later, tILC2s were isolated from resected donor-lungs and donor tumors. In recipient (WT C57BL/6) mice, the tumors were first established by s.c. injecting primary TC-1 tumor cells into the right flank (Day 0). On Day 1 after tumor establishment, the donor-derived tILC2s or PBS controls were adoptively transferred by intravenous (i.v.) injection into the tail vein of the recipient mice. The tumors were allowed to grow over the next 35 days.

**Figure 5:**
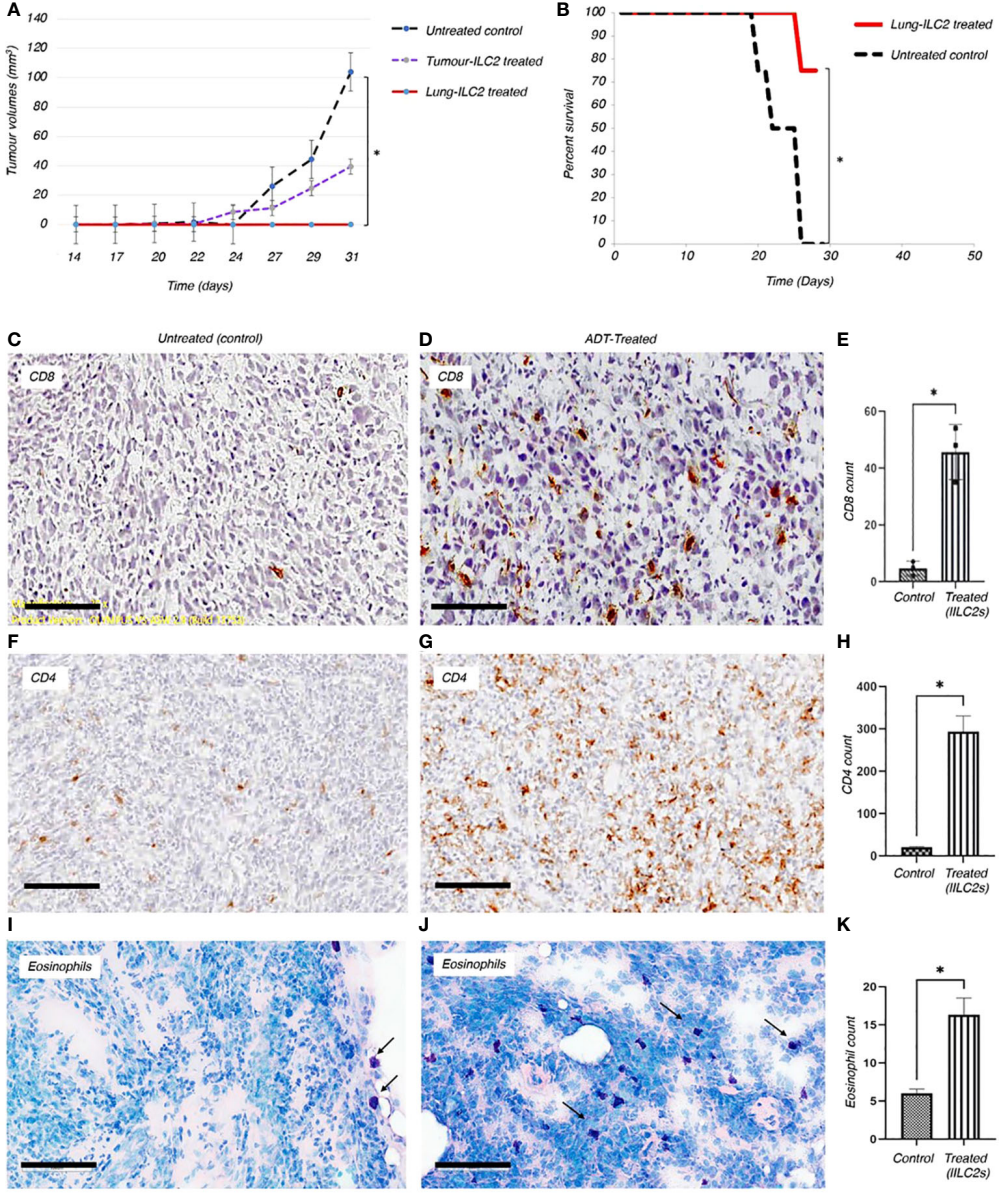
Adoptive transfer of ILC2s can reduce the growth of solid tumors and boost the anti-tumor immune responses *in vivo*. **(A)** Adoptive transfer of ILC2s into mice bearing TC-1 tumor transplants resulted in a significant decrease in overall tumor volume and reduced disease severity. ILC2s were isolated from either resected tumors or lungs of TC-1-tumour bearing donors. 1500 ILC2 cells from tumors or lungs were adoptive transferred into animals previously implanted with 5x10^4^ TC-1 tumor cells. The results were significant between animals treated with ILC2s and those with no ILC2 treatment; P<0.001 (Student’s T-test). Standard error bars have been added to all points. Paired T test: Control vs tumor ILC2s p=0.03; Control vs lung ILC2s p=0.008. ILC2s isolated from lungs were able to completely abrogate tumor growth. **(B)** In a separate experiment, 10,000 lung-derived ILC2 cells were adoptive transferred into animals previously implanted with 6x10^5^ TC-1 tumor cells. The overall survival rate for established mouse tumors was 75%, corresponding to data from eight representative animals in each group. Changes initiated by adoptively transferred ILC2s into mice bearing TC-1 tumor transplants resulted in a significant reduction of disease severity, such as formation of ulcers, development of infections, and rate of tumor growth (Independent T-test, *P < 0.001). **(C-K)** Changes initiated by adoptively transferred ILC2s into mice bearing TC-1 tumor transplants resulted in a significant increase of immune cells infiltration into tumor mass, when compared to untreated controls. Thus, the higher numbers of CD8^+^ **(C, D)** and CD4^+^ **(F, G)** T cells were indicated in brown. **(E, H)** The total number of CD8^+^ and CD4^+^ T cells infiltrated into a tumor mass of treated and untreated animals was quantified and p values were calculated accordingly, as p=0.0176, and p=0.0167. 5 μm thick sections were stained with appropriate antibodies and imaged at 20x magnification (Size bar = 200µm). **(I, J)** Eosinophils (dark purple) are located on the border between TC-1 tumor mass and normal tissue of the untreated animals. Adoptively transferred ILC2s allowed eosinophilic infiltration into the tumor tissue. **(K)** Quantification of eosinophils infiltrated into tumor mass between control and treated mice (p value is 0.0474). 10µm thick tumor sections were stained with Giemsa stain and imaged at 10x magnification (Size bar = 200µm).

A significant suppression of growth (100%) of the TC-1 tumors and reduced disease severity were observed in mice after adoptive transfer of tILC2s isolated from donor lungs (**Figure 5A**). The animals in this group had lower mean body weight loss, when compared to a control group, specific for the animal’s distress during cancer progression (data not shown). Interestingly, the tILC2s isolated from donors’ tumors were not as efficient, but still decreased the primary tumor volume by 60%, suggesting that tILC2 functionalities may be affected by the existence of phenotypic differences between tILC2 subsets that have to be studied in future experiments. In general, ILC2s isolated from lungs phenotypically have a higher number of ST2 receptors (35, 46–48) than ILC2s isolated from other organs, and, therefore, are able to respond rapidly to IL-33 stimulation, more significantly activating downstream signaling through ST2 receptors with consequent enhancement of cytokine-effector functions attracting more immune cells to the tumor site. More subtype validation and ADT experiments are underway to test this hypothesis.

### Adoptive transfer of tILC2s can improve the overall survival rate in mice bearing tumors

To investigate whether tILC2s can mediate the protective anti-tumor immunity for a larger tumor load, we established larger tumors using 12x times more TC-1 tumor cells (**Figure 5B**) than we used for tumor establishment in the previous experiment (**Figure 5A**). Here we adoptively transferred 10,000 activated tILC2s derived from lungs into each animal bearing a TC-1 tumor, and analyzed clinical characteristics of disease severity, such as formation of ulcers, development of infections, and a survival rate. We found that the overall survival rate for the tILC2 treated group was 75% (p<0.001) (**Figure 5B**). Adoptively transferring tILC2s into mice bearing TC-1 tumor transplants resulted in a significant reduction of disease severity and prevented formation of ulcers in the treated group. Whereas all animals in the control group reached the humane end point by Day 27, as they developed ulcerating cancers, the conditions when tumors growing under the skin break through the skin surface. These results suggest that adoptive transfer of a small number of tILC2s can mediate protective anti-tumor immunity for a large tumor mass. Thus, the transfer of tILC2s in the range of 1 tILC2 cell per ~ 60 tumor cells (10K/600K) can improve the overall survival rate in mice bearing tumor transplants. The higher number of tumor cells used to establish the described model is associated with a higher antigen load and, therefore, with more severe disease progression. More studies are on the way to optimize the treatment protocols using different subpopulations of tILC2 cells.

To gain deeper insights into the immune responses within the tumor microenvironment, we employed immunohistochemical staining techniques to visualize the immune cells infiltrating the tumors. The results revealed a striking contrast in the immune cell composition between the untreated control group and the adoptively-treated animals. In the tumors from the untreated controls, there were visibly fewer CD8^+^, CD4^+^ T lymphocytes and eosinophils that managed to penetrate the tumor mass (**Figures 5C, F, I**). This scarcity of infiltrating immune cells in the untreated tumors indicated a limited immune response within the tumor microenvironment. In stark contrast, in the tumors of animals which underwent ADT treatment using activated tILC2 cells, we detected a significantly higher number of leukocytes within the tumor tissue, as illustrated in **Figures 5C–K**, and it is well in line with our data provided in Xia *et al*. regarding the enhanced T cells recruitment abilities in activated tILC2s (39). This influx of CD8^+^, CD4^+^ T lymphocytes and eosinophils suggested a robust enrichment of both innate and adaptive immune response against the tumor cells in the treated animals. This comparative analysis highlighted the efficacy of the tILC2 adoptive transfer in enhancing immune cell infiltration within the tumor body, offering crucial insights for the development of immunotherapeutic approaches aimed at modulating the tumor microenvironment to promote effective immune responses against cancers.

### Eosinophilic infiltration into tumor tissue in IL-33 expressing murine lung and human prostate carcinomas

**Figure 6:**
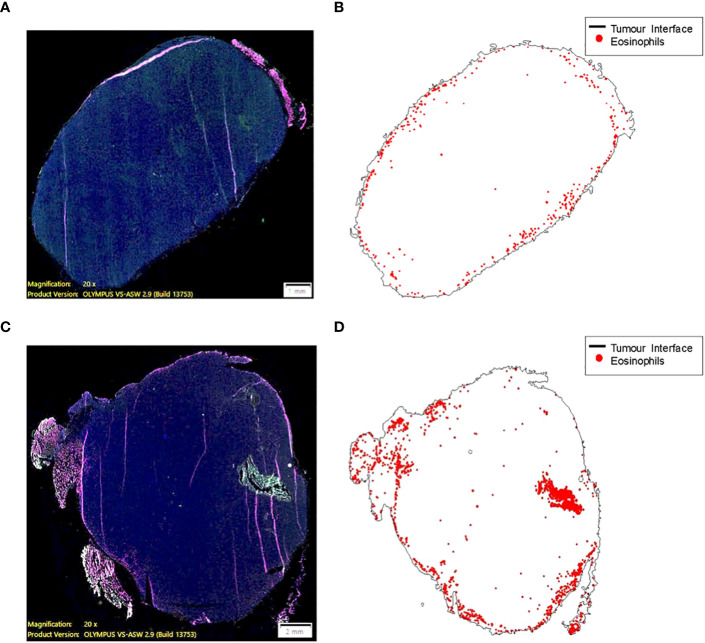
Eosinophil recruitment into tumor tissue is induced by the adoptive transfer of ILC2s (C57BL/6 mouse). **(A, B)** A small number of eosinophils (0.068% of total cell count) are mainly located on the border between TC-1 tumor and normal tissue. The higher eosinophil density, ~50 cells per 1mm^2^, along the surface of the tumor (less than 200 μm from a zero point). **(C, D)** ILC2-induced changes allowed a higher number of eosinophils (0.353% of total cell count) to be attracted to the tumor mass, as well as deeper penetration of cells (eosinophilic infiltration) into tumor tissue. The higher eosinophil density, >100 cells per 1mm^2^, >2500 μm away from the surface of the tumor and closer to the central part. 10 μm thick tumor sections were stained and imaged at 20x magnification (size bar = 1 mm **(A)**, size bar = 2 mm **(C)**.

The role of the eosinophils in anti-tumor immunity remains controversial , as their contribution is influenced by the tumor microenvironment and varies according to the disease stage (49). Our data demonstrates there are a greater number of tumor infiltrating eosinophils in tumor bearing animals adoptively transferred with tILC2s (**Figures 5I–K**). To test the effect of ADT-treatment on eosinophil recruitment into the tumor mass, we conducted a pathology study on mouse tumors before and after adoptive transfer of tILC2s. We detected an overall smaller number of eosinophils (**Figures 6A, B**) that were mainly located on the tumor surface in untreated control group. Whereas the ADT-induced changes allowed a higher number of eosinophils (**Figures 6C, D**) to be attracted deeper, closer to the center of the smaller tumor body, suggesting that tILC2-generated products could modify the tumor microenvironment. For instance, the heightened expression of IL-5 by activated tILC2s, as demonstrated in the single-cell RNA data analysis by Xia et al. (39) could have diverse impacts on eosinophils, potentially facilitating their infiltration into the tumor mass and thereby contributing to an anti-tumor effect. Thus, the adoptive transfer of tILC2s increased the eosinophilic infiltration into tumor tissue potentially contributing to protective anti-tumor immunity in murine lung carcinoma.

**Figure 7:**
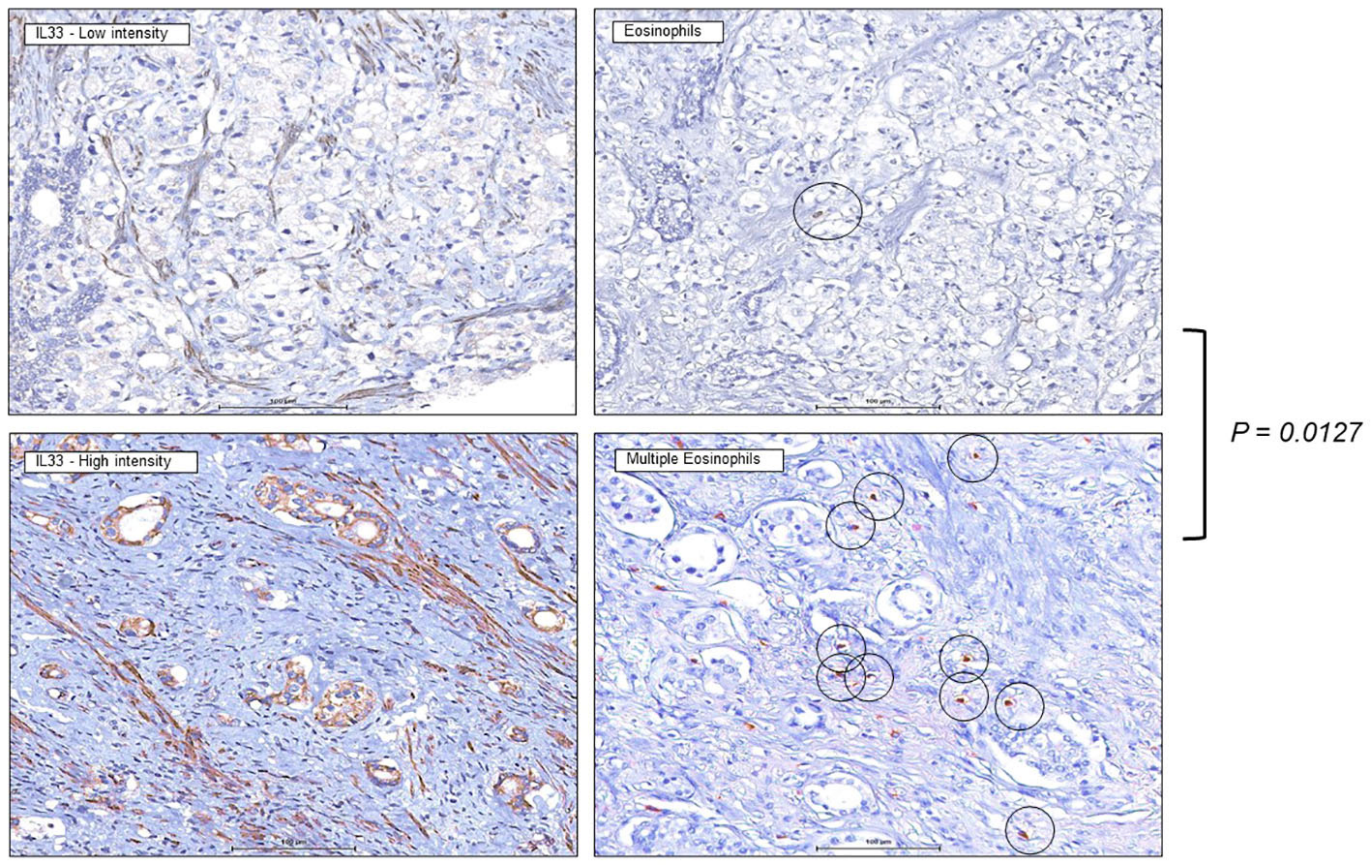
Increased eosinophils presence in human prostate tumors expressing IL-33. Immunohistochemistry staining was performed on human tissue microarrays (TMAs) of prostate cancers (PCa) expressing low levels of IL-33 (left), or high levels of IL-33 (right), indicating greater eosinophil number in IL-33 tumor sections. 4µm thick PCa TMA sections were imaged at 40X magnification, eosinophils are indicated by the circles. IL-33 staining intensity was scored as 0 (negative), 1 (low), or 2 (high).

We previously demonstrated correlation of IL-33 protein expression level with the biochemical recurrence of the human prostate tumors, which represent different stages of the human prostate cancer (27). Knowing that high IL-33 content is associated with initial stages of the disease progression and, at the same time, is favorable for ILC2 cell functionality, we performed an IHC study targeting eosinophils in human prostate tumors with high and low IL-33 content. Increased number of eosinophils in human tumor sections with higher IL-33 levels was detected (**Figure 7**), when compared to human tumor sections with lower IL-33 levels. This data suggests that eosinophils infiltration into IL-33-expressing tumors, may contribute to anti-tumor immune responses at earlier stages of human prostate carcinoma development, creating favorable background for ILC2 cells development and function, as well as for the future therapeutic applications related to IL-33/ILC2 axis in patients with human prostate tumors.

## Discussion

Cytotoxic T lymphocytes (CTLs), constitute a crucial component of the immune system, and play a pivotal role in cancer immunosurveillance (1). These specialized immune cells possess the remarkable ability to directly recognize and target tumor cells for destruction. Through the recognition of specific antigens present on the surface of tumor cells, CTLs are activated and mobilized into action (50, 51). Once activated, CTLs unleash their cytotoxic arsenal, which includes perforin and granzymes. Perforin forms pores on the target cell membrane, enabling the entry of granzymes, which induce apoptosis, a programmed cell death, in the tumor cell. This precise mechanism ensures the targeted elimination of tumor cells, making CTLs an essential weapon in the body’s defense against cancer. By directly killing and specifically eliminating tumor cells, CTLs contribute significantly to the body’s immune response, highlighting their critical role in immunosurveillance and potential applications in cancer immunotherapy (50, 51).

The foundation of the present studies rested upon our initial discovery and subsequent validation of the association between IL- 33 and cancer immune surveillance (1, 27), and the role that ILC2s play in anti-tumor Th1 immunity and promoting CTL function (26). Our previous studies led to the discovery of a direct role of ILC2s in Th1-related responses, such as ILC2-dependent CTLmediated immune surveillance against tumors, or uncontrolled growth of tumors in mice that lacks ILC2 cells (RORa-/- mice) (26). In particular, the presence of ILC2s significantly inhibited tumor formation in WT chimeric mice bearing tumors expressing IL-33, when compared to RORa−/− chimeras, suggesting the role of IL-33/ILC2s axis in mediating the anti-tumor immunity. A direct link between ILC2s and CD8+ CTLs was demonstrated by observing elevated levels of granzyme B and increased killing of tumor cells by CTLs during co-culture in the presence of ILC2s but not in their absence (26). We also showed that CTL effector function is heightened in the presence of tILC2s (26). This pivotal discovery demonstrated that IL-33-dependent ILC2s play a functional role in IL-33-mediated anti-tumor immunity (26). This research began to unravel the intricate relationship between IL-33, ILC2s, and their collective impact on the body’s ability to combat cancer. By exploring the functional dependencies and interactions within this complex immune system pathways, these studies shed light on novel avenues for enhancing anti-tumor immune responses and advancing cancer immunotherapy strategies. Thus, our previous studies demonstrated for the first time that ILC2s aid Th1 responses by facilitating the interactions between the innate and adaptive arms of immune response.

In this study, we extend our previous studies on the importance of ILC2s in promoting Th1-dependent CTL anti-tumor responses and thereby make several novel observations related to the potential of IL-33/ILC2 axis to confer immune protection against tumors. We demonstrate the effect of a growing tumor on the tILC2s protein production level. We confirm our earlier discovered inverse correlation between the IL-33/ILC2 alignment and the tumor growth pattern, conducting an *in vivo* ADT study, utilizing the advantage of tILC2 cell-plasticity. These findings underscore the potential of modulating the tumor microenvironment as a powerful strategy in cancer immunotherapy.

Here we also demonstrate that mice lacking CTLs or T helper cells have higher tumor burdens (**Figure 1**), whereas the *GATA1^-/-^* group, lacking eosinophils, showed smaller average tumor volume but not significantly different to the WT group with complete immune cell context. The presence of a comprehensive immune landscape compensates for eosinophil deficiency, which may otherwise contribute to the control of tumor progression. Notably, through ADT of additional ILC2s, eosinophils gain the capability to infiltrate the tumor mass and exert stronger effector functions (**Figures 5**, **6**). Eosinophils are a rare, blood-circulating leukocyte population that is known to be involved in host protection against parasites and inflammation, providing the first line of defense and initiation of downstream inflammatory immune responses (52). The role of eosinophils is functionally linked to the presence of IL-33 in the microenvironment and has been controversial regarding anti-tumor immunity, which largely depends on the tumor type and the immune cell populations within the microenvironments (49). Our clinical study on human prostate tumors (**Figure 7**) demonstrates the direct correlation between eosinophilic infiltration and IL-33-expression, suggesting the contribution of the IL-33/ILC2 axis to anti-tumor immune responses at earlier stages of human prostate carcinoma. This discovery provides the impetus for future therapeutic applications related to IL-33/ILC2 axis in patients with human prostate tumors. More studies are required to address the contribution of IL-33/ILC2s/eosinophils alliance to the immune response in various forms and stages of the prostate neoplasms.

Given the existence of remarkable similarities between ILC2s and CD4^+^ T helper lymphocytes, we hypothesized that the adoptive transfer of tILC2s could reduce the growth of tumors and demonstrated, for the first time, the direct involvement of tILC2s in immunologically limiting tumor growth through the recruitment of key immune players including CD4^+^, CD8^+^ T cells, and eosinophils. We also identified how a common set of transcriptional regulators (shared by developmental programming of ILC2s and T lymphocytes) may enable tILC2 to acquire unique effector functions. The approach highlighted here may have practical applications for cell-based immunotherapy, as adoptively transferred tILC2 cells confers immune protection from tumors at lower numbers (1 tILC2 cell per 30-60 tumor cells) to those reported for adoptively transferred CAR-T cells and autologous T cells (53).

Our previous study (39) demonstrated the ILC2-plasticity during tumor progression on scRNA level, as an acquisition of the new type-1-like immune functions. In the current study, we provide the analysis a set of 32 cytokines and chemokines proteins secreted by tILC2s during neoplasm development, when compared to the protein profile of nILC2 cells. We also observed the lower expression of Gata3 gene in tILC2s than in the naïve counterparts (**Figure 3**). Given the importance of Gata3 in ILC2 development and function (54), it is now well established that down-regulation of this transcription factor is a common trend in ILC2 identity shifts (40, 55–58). Gata3 gene was down-regulated in parallel with the most striking and the most significant up-regulation in I*l*-12 (p70) expression. The full length of biologically active IL-12 (p70) was secreted by tILC2s, but not by nILC2s, suggesting that it can drive the cytokine production plasticity (40), as well as promote the effector function of CD8^+^ T lymphocytes in an IL-33 expressing environment (42). In parallel with considerable overproduction of other pro-inflammatory modulators, such as TNFα, IL-2, IL-6, MIG (CXCL9), MIP-1β (CCL4) and MIP-2 (CXCL2), we also detected a significantly lower secretion of IL-10 by tILC2s that contradicted the conventional ILC2 role in cancer (22, 23). Thus, a simultaneous suppression of the type-2-related mediators and overexpression of pro-inflammatory factors, type-1-like mediators, implies that these paracrine interactions, stimulated by a growing tumor, allow cells to create a network of communications that is suitable for the immunological tasks, of aiding immune cell growth, recruitment and activation (44). Therefore, the growing neoplasm promotes cytokine production plasticity in tILC2 cells that has the knock-on effect of promoting Th1 responses.

This study deepens our understanding of the complex dynamics within the tumor microenvironment by demonstrating *in vivo* the earlier discovered inverse correlation between the number of ILC2s and tumor size in the IL-33-treated animals (**Figure 4C**). This intriguing observation, where smaller tumors harbor a higher count and frequency of ILC2s in response to IL-33 treatment, marks a significant departure from the conventional understanding of tumor-immune interactions. This phenomenon, unique to the IL-33-treated mice and absent in the control group treated with PBS, defines a specific role for ILC2s in regulating immune surveillance and tumor development. The increased presence of ILC2s in smaller tumors suggests its suppressive effect on tumor growth, indicating a potential mechanism by which these immune cells contribute to restricting tumor expansion (**Figure 4**). This finding has far-reaching implications, particularly in the context of cancer immunotherapy. Understanding the intricate interplay between the immune response, cytokine treatments like IL-33, and tumor progression is crucial for developing targeted and effective immunotherapies. The observation highlights the complexity of immune regulation within the tumor microenvironment and emphasizes the need for further exploration. Moreover, the discovery of this inverse correlation opens avenues for exploring novel therapeutic strategies. Harnessing the potential of IL-33/ILC2s axis and understanding the mechanisms underlying their suppressive effect on tumor growth could pave the way for innovative immunotherapeutic approaches.

On the functional level, we show that the adoptive transfer of tILC2, which possess enriched pro-inflammatory and T lymphocyte recuritment power, significantly reduces tumor growth rate (**Figure 5A**), and improve the survival rate among animals bearing larger tumors (**Figure 5B**). Specifically, the transfer of tILC2s heightens the recruitment of both innate and adaptive immune responses by recruiting CD8^+^ T lymphocytes, CD4^+^ T lymphocytes, and eosinophils into the tumor mass (**Figures 5C, K**), thereby highlighting the potential of tILC2s boosting the immune response to tumors, and therefore, providing a generalized approach to cancer immunotherapy. Furthermore, the visual evidence provided by immunohistochemical staining is pivotal. In the untreated control group, the scarcity of CD8^+^, CD4^+^ T lymphocytes, and eosinophils penetrating the tumor mass indicated a limited immune response within the tumor microenvironment (**Figures 5C, F, I**). In contrast, after ADT-tILC2 treatment, animals exhibited a significant increase in infiltrating CD8^+^, CD4^+^ T lymphocytes and eosinophils (**Figures 5D, E, G, H, J, K**), specifying a robust and targeted immune response against the tumor cells. Additionally, the altered density of the treated tumor mass implies significant changes within the tumor microenvironment due to the adoptive treatment. This change could involve cell death, altered cellular composition, or other processes that are yet to be fully understood.

To further elucidate the immune landscape functionally linked to the IL-33 expressing environment, we conducted an immunohistochemistry (IHC) study targeting eosinophils in human prostate tumors characterized by high and low IL-33 content. The results revealed a discernible increase in the number of eosinophils in tumor sections exhibiting higher IL-33 levels compared to those with lower IL-33 levels, (**Figure 7**). This observation suggests a potential link between eosinophil infiltration and IL-33-expressing tumors, indicating that eosinophils may contribute to an anti-tumor immune response, particularly in the earlier stages of human prostate carcinoma. The increased presence of eosinophils in tumors with higher IL-33 levels implies a supportive microenvironment for the development and function of ILC2s. Given the known favorable role of ILC2s in immune responses, this association suggests a complex interplay involving IL-33, eosinophils, and ILC2s in the context of human prostate tumors.

Overall, the scientific evidence provided in this study lays a foundation for future investigations aiming to leverage the IL-33/ILC2 axis for therapeutic interventions in cancer. There are several advantages of the adoptive transfer of tILC2s, when compared to other anti-cancer T lymphocyte therapies, such as using CAR T-cells. For example, the experimental timing of the adoptive transfer of immune cells is shorter. In the case of CAR T-cell based approaches, T lymphocyte preparation *in vivo* takes a minimum of 5 days. Moreover, lympho-depletion via radiation is required prior to cell transfer (59). Furthermore, the ratio of adoptively transferred tILC2s to tumor cells in our studies was on the order of one ILC2 cell to ~30-60 tumor cells, whereas in models of human tumor grown in NSG mouse, the ratio of CAR positive T lymphocytes to tumor cells inverses this relationship, and was on the order of ~5-10 CAR positive T lymphocytes to one tumor cell (59). For example, Klampatsa et al. (58) used the same TC-1 tumor model we used in this study to demonstrate that a ratio of 5 CAR-T cells to 1 TC-1 tumor cells to achieve a modest reduction of TC-1 tumors grown subcutaneously (53). Confirming this in the wider CAR-T lymphocyte field, in models of human tumor grown in NSG mouse, the ratio of CAR+ T lymphocytes to tumor cells is on the order of ~5-10 CAR+ T lymphocytes to 1 tumor cell as well (59). Therefore, when compared to similar studies involving adoptively transferred CAR positive T lymphocytes, tILC2s appear to be a minimum of 150 times more effective on a stoichiometric basis compared to adoptively transferred CAR positive T lymphocytes in promoting tumor-free survival. Furthermore, there is the possibility of using this approach on patients without regard to antigen specificity that again is a significant advantage compared to other cell-based therapies. Overall, compared to CAR-T lymphocyte approaches, the efficiency of the adoptively transferred tILC2s is impressive. However, future studies are required to optimize the tILC2 adoptive transfer protocol and expand on the comparison of ILC2s isolated from different organs of WT-donor and tumor-bearing donor mice. Moreover, adoptively transferred ILC2 treatment can be developed in combination with other anti-cancer therapeutic approaches, such as in combination with immune checkpoint blockade therapies (biologics against PD-1, CTLA-4 or OX40), cytokines such as IL-33, or radio- and chemotherapies to enhance the outcomes. We conclude that the adoptive transfer of tILC2s significantly reduces the growth of tumors and underlines their potential utility in novel cell-based anti-cancer immunotherapeutic approaches.

## Materials and methods

### I) *In vitro* studies

#### Cell lines: murine lung cancer model

The TC-1 cell line is a murine lung tumor model derived from primary lung epithelial cells of C57BL/6 mice immortalized using the amphotropic retrovirus vector LXSN16 carrying human papillomavirus (HPV) genes E6/E7, and subsequently transformed with pVEJB plasmid expressing the activated human c-Ha-ras oncogene (60). TC-1 cells display high expression of TAP-1, MHC-I, and IL-33 (61). The metastatic cell line A9 was derived from the TC-1 tumor cell line and displays spontaneous down-regulation of MHC-I (or H-2 Histocompatibility Class I genes) and IL-33 by immunoselection *in vivo* during immunization/challenge experiment (62, 63). Therefore, they are a unique model to study the role of ILC2s in tumor growth. The cell line was grown in Dulbecco’s modified Eagle medium, supplemented with 10% heat-inactivated fetal bovine serum, 2 mM l-glutamine, 100 U/ml penicillin, 100 μg/ml streptomycin, and 10 mM HEPES, at 37°C, with the air supplemented with 5% CO_2_. The cells were tested by IDEXX RADIL and found to be free of any contaminating viruses or mycoplasma.

#### Cytokine production assays

Day 3 StemSpan™ SFEM II culture supernatants from ILC2s were sent to Eve Technologies in Calgary, AB, for a Mouse Cytokine Array/Chemokine Array 32-Plex concentration analysis (MD31). https://www.evetechnologies.com/product/mouse-cytokine-array-chemokine-array-31-plex/

### II) *Ex vivo* studies

#### ILC2 isolation

Lungs or tumor tissues from donor-mice were harvested and cut into small pieces using a razor blade and digested for 30 minutes at 37°C in a shaker platform (200rpm) in 10mL of digestion media per 5 pairs of lungs. The digestion medium employed contained RPMI 1640 (Gibco #11875-093) with 100 U P/S and 10% FBS, as well as 1mL collagenase/hyaluronidase and 1.5mL DNase I per 5 pairs of lungs (StemCell #07912 and #07900 respectively).

The digested pieces of lung tissue were placed onto a 70µm cell strainer. Using the plunger end of a 3mL syringe, the tissue was mashed through the strainer and rinsed with 5mL RPMI 1640 to a total of 15mL. Cells were centrifuged for 6 minutes at 1600-1700rpm. The supernatant was carefully removed, and the pellet was re-suspended in 20mL ammonium chloride solution (StemCell #07800) and incubated at room temperature for 5 minutes to lyse the erythrocytes. After neutralization with 30mL of FACS buffer cells were counted and washed (6 minutes, 1600-1700rpm) in a total volume of 50mL (full 50mL Falcon Tube). FACS buffer was made of DPBS (Gibco #14190-136) with 2% FBS.

After counting cells, they were re-suspended in the appropriate volume of FACS buffer (PBS +2% FBS) to obtain 1X cells/mL, and then they were enriched for ILC2s using an EasySep™ Mouse ILC2 Enrichment Kit (StemCell #19842), which reduces sorting time and increases ILC2 recovery both from naïve and IL-33–treated lungs (64).

Prior to sorting, the cells were stained with FITC-conjugated lineage marker mouse antibodies purchased from Thermo Fisher Scientific (CD3ϵ/γ #11-0032-80, CD4 #11-0042-81, CD8α #11-0081-81, CD19 #11-0193-81, TCRβ #11-5961-81, NK1.1 #11-5941-81, TER119 #11-5921-81, CD11c #11-0114-81, CD11b #11-0112-41 and Ly-6G/C #11-9668-80) and the ILC2 positive markers purchased from BioLegend: PE-conjugated CD127 (IL-7 receptor, #135009), PerCP-Cy5.5-conjugated ST2 (IL-33 receptor, #145312), BV605-conjugated Thy1.2 (#140317) and BV421-conjugated CD45 (#103133).

#### Flow cytometry

BD FACS Aria II machine was used to phenotypically assess and sort the cells. Antibodies used to block Fc receptors: CD16/32 (564220, BD Pharmingen). Antibodies used to exclude nonviable cells from flow cytometry: Fixable Viability Dye eFluor 780 (65-0865-14, Affymetrix eBioscience).

#### Intracellular staining for flow cytometry

ILC2s derived from TC-1 tumor-bearing mice (tILC2s) were fixed and permeated using an intracellular fixation and permeabilization buffer set (eBioscience #88-8824-00) and then stained in PBS (2% FBS) with the following mouse antibodies: PE-conjugated granzyme B antibody (BioLegend #372207) and APC-conjugated granzyme C (BioLegend #150803). The IFITM1 mouse monoclonal antibody was purchased from Proteintech (#60074-1-Ig) and conjugated with a FITC antibody labeling kit (Mix-n-Stain #92295). The CD3γ mouse monoclonal antibody was purchased from Bioss Antibodies (#bsm-54300R) and conjugated with an APC-CF 750T antibody labeling kit (Mix-n-Stain #92311A). Both antibodies were conjugated at The University of British Columbia Antibody Laboratory.

### III) *In vivo* studies

#### Mice

C57BL/6, CD8-/-, CD4-/-, *GATA1-/-* (gifted from the lab of Dr. Avery August), C57BL/6-Tg(CAG-EGFP)1Osb/J and C57BL/6-Tg(CAG-EGFP)131Osb/LeySopJ mice were purchased from the Jackson Laboratory or gifted and maintained in the Centre for Disease Modeling at the University of British Columbia. Female mice were used at 4–8 weeks of age. These experiments were approved by the Animal Care Committee (UBC). Animals were maintained and euthanized under humane conditions in accordance with the guidelines of the Canadian Council on Animal Care.

#### IL-5 and IL-13 cytokines assays and cytokine array.

Freshly isolated ILC2s (~2x10^3^) cells 200ml/per well of a 96 w/plate) were re-stimulated *in vitro* for 3 hr at 37C using re-stimulation medium (with no cytokines added), as suggested by the Protocol published in 2014 by Halim et al. (65). A supernatant (100ml) was used for the IL-5 and IL-13 secretion analysis according to the manufacturer protocols: Mouse IL-5 Uncoated Enzyme-linked immunosorbent assay for quantitative detection of mouse IL-5 Catalog Number 88-7054; Mouse IL-13. Uncoated Enzyme-linked immunosorbent assay for quantitative detection of mouse IL-13 Catalog Number 88-7137. The cytokine was Mouse Cytokine/Chemokine 32-Plex Discovery Assay^®^ Array (MD32) from Eve Technologies (Calgary, Alberta) and was performed according to the suppliers specifications.

#### Tumor establishment

For implantation models, 5x10^5^ TC-1 cells or 1x10^4^ A9 cells or 6x10^5^ TC-1 tumor cells [in 50µl of HBSS (ThermoFisher Scientific)] were injected into wild type (WT) animals subcutaneously (s.c.) into the right flank (4-8 weeks old) (26, 27, 66). Tumor growth was monitored by measuring tumor dimensions using callipers. Tumor length and width measurements were obtained three times weekly. Tumor volumes were calculated according to the equation tumor volume=length x width x height x π/6 with the length (mm) being the longer axis of the tumor. Animals were weighed at the time of tumor measurement. Mice were euthanized if they reached a humane end point, based on 20% reduction in body weight, a tumor volume larger than 1 cm^3^ or ulceration of the tumor. The normal course of tumor growth *in vivo* varies depending on the cell line: 10-14 days for A9; ~25-35 days for TC-1 (26).

For preliminary nILC2 versus tILC2 study, to maximize ILC2 differentiation and harvest, all donor animals were intraperitoneally injected with recombinant mouse IL-33 (2ng/µL) (R&D Systems #3626-ML-010) three times every second day prior to ILC2 isolation.

#### Measuring ILC2 infiltration into tumors

Two strains of GFP+ mouse strains were used due to availability, C57BL/6-Tg(CAG-EGFP)1Osb/J and C57BL/6-Tg(CAG-EGFP)131Osb/LeySopJ. Both strains are highly similar, with widespread expression of EGFP in all cells except erythrocytes and hair, under the chicken beta-actin promoter and cytomegalovirus enhancer. TC-1 are injected s.c. to the right flanks of mice at 5x10^5^ cells per mouse on Day 0. Mice are treated with 200ng of rmIL-33, or PBS through i.p. injections on Day 7, 9 and 11. Animal handling follows the Animal Care and Use Program protocols at UBC. On terminal day at Day 18, lungs and tumors are collected individually from each mouse and processed for flow cytometry experiment. Lung enrichment follows the method previously described. Tumor processing includes the careful removal of attached skin, muscle, and tissue linings, leaving only the tumor mass, followed by weighing of each tumor individually. All the collected and enriched cells are subjected to flow cytometry analysis. Flow cytometry follows method previously described for FACS, except lineage markers are moved to APC channels to avoid overlapping with GFP signals.

#### Adoptive transfer

Donor C57BL/6 mice (tILC2-donor mice) were first primed with primary murine lung carcinoma tumors (TC-1), injected s.c. into the right flank (as described in the Tumor Establishment section). Approximately three weeks later, tILC2 cells were purified from donor-lungs or donor-tumors by FACS, as per ILC2 isolation protocol.

*Ex vivo* stimulation: the purified tILC2 cells were then cultured in RPMI-1640 media containing 10% FBS, P+S, 2ME and stimulated by IL-33 (10 ng/ml), IL-2 (10ng/ml) and TSLP (10 ng/ml) for 5 days.

Recipient C57BL/6 mice were injected Subcutaneous (s.c.) a dose of 50 µL of 5x10^4^ TC-1 tumor cells into the right flank (Day 0). On Day 1, after *ex vivo* stimulation, expanded tILC2 cells (1.5x10^3^ cells in HBSS) or HBSS control were injected via tail vein into each recipient animal. Tumor growth in recipient mice was monitored for up to 35 days. For each donor-, control- and treatment-group we used 6-8 female mice at 4-8 weeks of age.

#### Survival study

For tumor establishment on a recipient mouse, 6x10^5^ cells TC-1 tumor cells [in 50µl of HBSS (ThermoFisher Scientific)] were injected into wild type animals subcutaneously (s.c.) into the right flank (4-8 weeks old) on Day 0. On Day 1, after *ex vivo* stimulation, expanded donor-lungs-tILC2 cells (1x10^4^ cells in HBSS) or HBSS control were injected via tail vein into each recipient animal (bearing 6x10^5^ of TC-1-tumour-cells). Clinical characteristics of disease severity were observed for 30 days or until reaching humane endpoint.

#### Mouse study: immunohistochemistry staining for CD8^+^T cells, CD4^+^ T cells, eosinophils

Frozen samples were submitted to The Centre for Phenogenomics (Toronto, Canada) for processing. Sample blocks were cut on a CryoStar NX70 cryostat (Thermo Scientific) into 8-μm tissue sections and collected on charged microscope slides (Assure, Epic Scientific). These tissue sections were fixed in 10% neutral buffered formalin (Fisherbrand, cat # 245-685) and permeabilized with 0.5 v/v% Triton X-100 (Sigma, cat # T8787) in 1× PBS. The section was then incubated in Protein Block (Agilent, cat # X090930-2) to minimize non-specific antibody binding. Primary antibodies were diluted in Antibody Diluent (Agilent, cat. no. S302283-2) overnight at 4°C. The primary antibodies used in this study were Rabbit anti-CD4 (Abcam, cat # ab183685, 1:250) and rat anti-CD8 (Invitrogen, cat # 14-0081-82, 1:100). The next day, the primary antibody solution was removed, and the tissue section was washed in 1× tris-buffered saline (Diamed, cat # DIABUFFER-TBS) with 0.1 v/v% Tween-20. Fluorophore-conjugated secondary antibodies were diluted in Antibody Diluent and the tissue sections were incubated for 1h at room temperature. The secondary antibodies used in this study were Anti-Rat IgG + Alexa Fluor 488 (Invitrogen,cat # A11006, 1:200) and Anti-Rabbit IgG + Alexa Fluor 750 (Invitrogen, cat # A21039, 1:100). After washing in buffer and deionized water, phenol red histochemistry staining for detection of eosinophils was performed based on the protocol described by Ain et al. (DOI: 10.1016/s0022-1759 (01)00526-9). The slides were immersed in Hank’s Balanced Salt Solution with 56 uM phenol red (Wisent, cat # 311-511-CL) and incubated with gentle rocking for 2 hours at room temperature. The sections were rinsed in Hank’s Balanced Salt Solution (Wisent, cat # 311-512-CL), then counterstained with DAPI (Sigma, cat # D9542). Autofluorescence from tissue was quenched in Sudan black B saturated solution in 70% ethanol (Sigma, cat # 199664) for 25 minutes at room temperature. Finally, coverslips were mounted with Vectashield Vibrance anti-fade mounting medium (Vector; cat # H-1700). Slides were scanned at 20X using an Olympus VS120 slide scanner equipped with a Hamamatsu ORCA-R2 C10600 digital camera (Evident, Tokyo, Japan). Image analysis of the scanned slides was performed with the HALO Image Analysis Platform V3.6.4134 (Indica Labs, USA), using the Highplex FL V4.2.14 module for quantification and spatial infiltration analysis of phenol red-positive eosinophils.

#### Human study: immunohistochemistry staining of human tissues for IL-33 and eosinophils

Prostate cancer (PCa) tissue microarrays (TMAs) were obtained from the Vancouver Prostate Centre at the University of British Columbia. All patients had provided informed consent for their tissues to be used for research purposes (UBC Clinical Research Ethics Board # H09-01628). The cohort selection detail was described previously (PMID: 24052624). Briefly, the cancer regions were selected, based on the highest tumor content, by a research pathologist (L.F) and all the selected areas were marked and punched for a TMA construction. 4µm thick sections of PCa TMAs were used for IHC staining using the Ventana DISCOVERY Ultra. For IL-33 staining, the antigen retrieval was conducted with CC1 at 64 min, then the primary antibody (HPA024426, Sigma-Aldrich, 1:50 dilution) was incubated for 12 hours at room temperature. IL-33 signal was detected with DAB Map Detection Kit (Ventana). For eosinophil staining, the antigen was retrieved with Protease 2 (Ventana) at 8 min, then the primary antibody (NBP1-42140, Novus Biologicals, 1:50 dilution) was incubated for 2 hours at room temperature. Eosinophil signal was detected using UltraMap DAB anti-Ms Detection Kit (Ventana). All stained slides were digitized with Leica scanner (Aperio AT2, Leica Microsystems; Concord, Ontario, Canada) at magnification equivalent to 40X. The images were subsequently stored in the Aperio eSlide Manager (Leica Microsystems) at the Vancouver Prostate Centre. The IL-33 staining intensity was scored as 0 (negative), 1 (low) or 2 (high). For eosinophils, manual counting was done and grouped as low (0-5 positive cells), moderate (6-20 positive cells) and high (≥21 positive cells).

#### Statistics

Data were analyzed with R, Excel, and Graphpad Prism. A Student’s t test was used for determining statistical significance between groups in ADT; p ≤ 0.05 was considered significant.

## Data availability statement

The raw data supporting the conclusions of this article will be made available by the authors, without undue reservation. Data was made available as a preprint and can be found on the following link: http://dx.doi.org/10.2139/ssrn.4104818 (Saranchova, Iryna and Xia, Clara Wenjing and de Lucía Finkel, Pablo and Munro, Lonna and Pfeifer, Cheryl G. and Jefferies, Wilfred, A Novel Cell-Based Immunotherapy Utilizing Adoptive Transfer of Type-2 Innate Lymphoid Cells to Reduce the Growth of Tumours. Available at SSRN: https://ssrn.com/abstract=4104818 or http://dx.doi.org/10.2139/ssrn.4104818).

## Ethics statement

The animal study was approved by Animal Care Committee University of British Columbia. The study was conducted in accordance with the local legislation and institutional requirements.

## Author contributions

IS: Conceptualization, Formal analysis, Investigation, Methodology, Writing – original draft, Writing – review & editing. CX: Formal analysis, Investigation, Writing – original draft, Writing – review & editing. SB: Formal analysis, Investigation, Writing – review & editing. PLF: Formal analysis, Investigation, Writing – original draft. SLSE: Formal analysis, Methodology, Validation, Writing – original draft. SK: Formal analysis, Investigation, Methodology, Writing – review & editing. LM: Formal analysis, Investigation, Methodology, Writing – review & editing. CGP: Conceptualization, Formal analysis, Writing – review & editing. LF: Methodology, Project administration, Visualization, Writing – review & editing. MG: Methodology, Project administration, Supervision, Writing – review & editing. WAJ: Conceptualization, Formal analysis, Investigation, Methodology, Supervision, Writing – original draft, Writing – review & editing.
