# Supplementary material for: A novel type-2 innate lymphoid cell-based immunotherapy for cancer
Source: Front Immunol. 2024 Mar 7;15:1317522. doi: 10.3389/fimmu.2024.1317522 (PMC10958781; doi:10.3389/fimmu.2024.1317522)
Supplement: Supplementary file 1 [file DataSheet_1.docx]

**Appendix:**

**
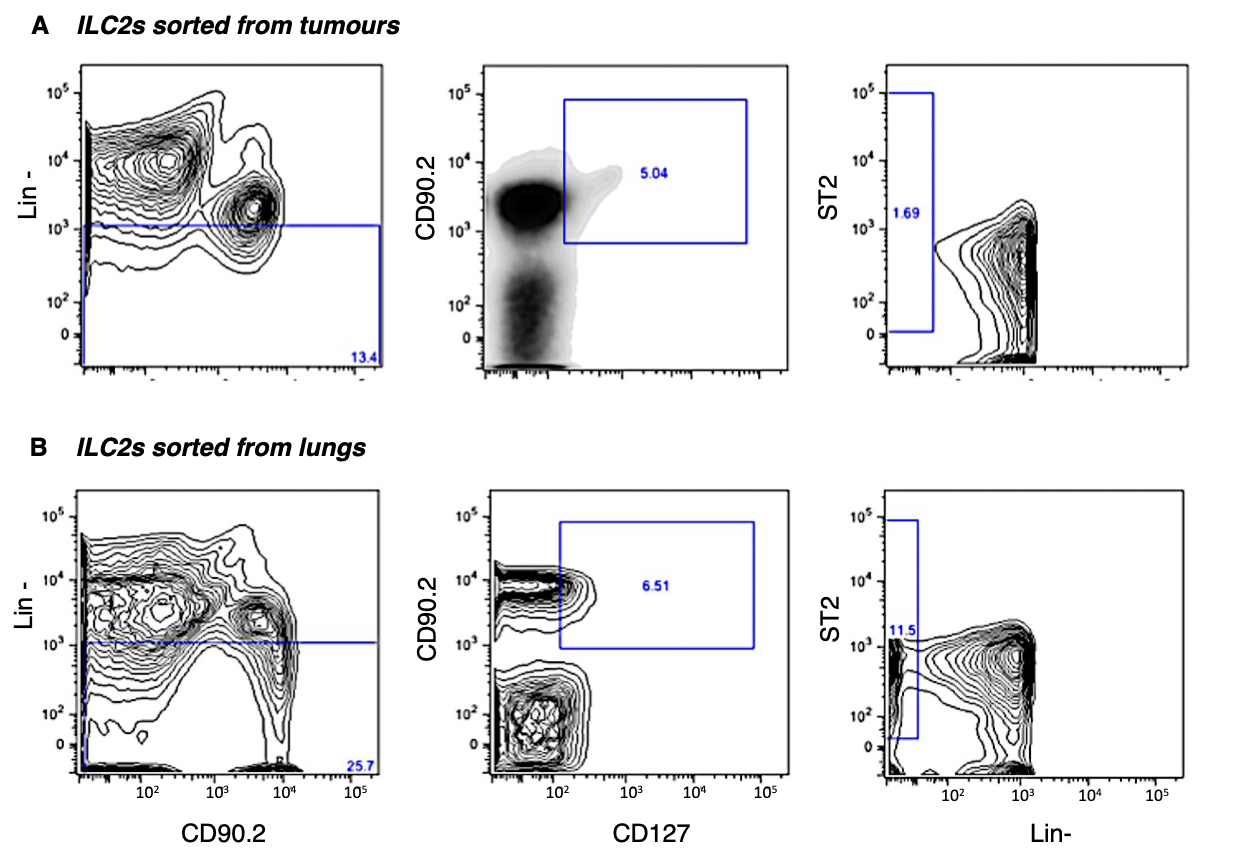
**

**Supplementary Figure 1. ILC2 cells isolation and purity validation for ADT experiment.** **(A)** ILC2 cells sorted from lungs. **(B)** ILC2 cells sorted from tumours. Cells that expressed FITC-conjugated lineage-related markers (such as CD3, CD4, CD8α, CD19, TCRβ, NK1.1, TER119, CD11c, CD11b, Ly-6G/C) were rigorously depleted during the isolation process. ILC2 cells were finally isolated based on CD127, ST2, Thy1.2 positive expression from the ST2+/Lin- gate, where ST2-marker was plotted versus lineage markers to enhance the purity of the selected cell population.

**
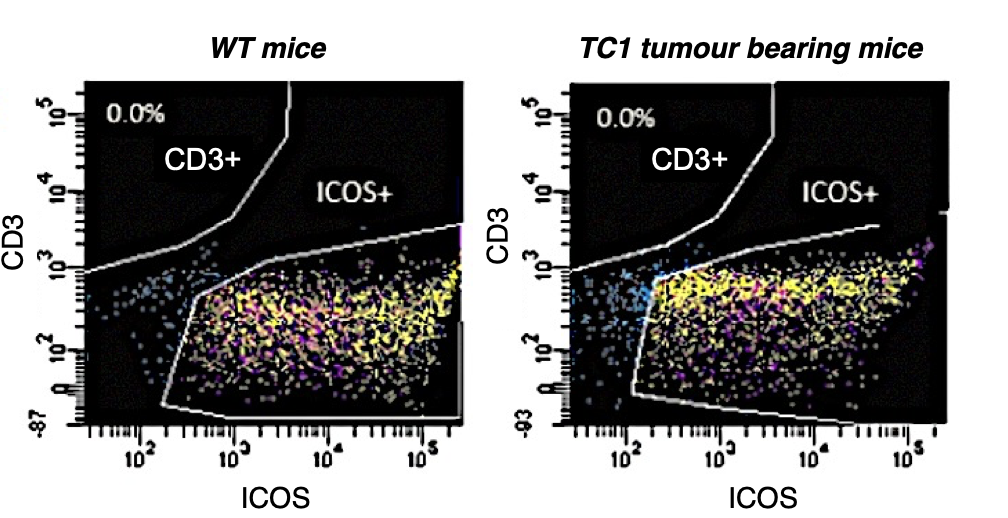
**

**Supplementary Figure 2. ILC2 cells isolation and purity validation for ADT experiment.** Cells that expressed FITC-conjugated lineage-related markers (such as CD3, CD4, CD8α, CD19, TCRβ, NK1.1, TER119, CD11c, CD11b, Ly-6G/C) were rigorously depleted during the isolation process. ILC2 cells were finally isolated based on CD127, ST2, Thy1.2 positive expression from the ST2+/Lin- gate, where ST2-marker was plotted versus lineage markers to enhance the purity of the selected cell population. The purity of isolated ILC2 cells, and the absence of T-cell contamination in culture, during *in vitro* activation processes, were validated by the absence of CD3 marker expression at various time points using flow cytometry.

**Supplementary Figure 3**- Isolation of EGFP+ ILC2s from lungs

**Supplementary Figure 4**- Isolation of EGFP+ ILC2s from tumours

**Supplementary Table 1** - List of antibodies used to isolate and characterize ILC2s

| Antibodies used |  |
| --- | --- |
| APC | CD3e/y |
|  | CD4 |
|  | CD8a |
|  | CD19 |
|  | TCRb |
|  | NK1.1 |
|  | TER119 |
|  | CD11c |
|  | CD11b |
|  | Ly-6G/C |
|  |  |
| PE | CD127 |
| PerCP-Cy5.5 | ST2 |
| BV605 | Thy1.2 |
| BV421 | CD45 |
